# Morphology-Controlled Vapor Phase Growth and Characterization of One-Dimensional GaTe Nanowires and Two-Dimensional Nanosheets for Potential Visible-Light Active Photocatalysts

**DOI:** 10.3390/nano11030778

**Published:** 2021-03-18

**Authors:** Li-Chia Tien, Yu-Che Shih

**Affiliations:** Department of Materials Science and Engineering, National Dong Hwa University, Shoufeng, Hualien 974, Taiwan; 610622007@gms.ndhu.edu.tw

**Keywords:** 1D, 2D, GaTe, physical vapor transport, visible-light active photocatalysts

## Abstract

Gallium telluride (GaTe) one-dimensional (1D) and two-dimensional (2D) materials have drawn much attention for high-performance optoelectronic applications because it possesses a direct bandgap for all thickness. We report the morphology-controlled vapor phase growth of 1D GaTe nanowires and 2D GaTe nanosheets by a simple physical vapor transport (PVT) approach. The surface morphology, crystal structure, phonon vibration modes, and optical property of samples were characterized and studied. The growth temperature is a key synthetic factor to control sample morphology. The 1D GaTe single crystal monoclinic nanowires were synthesized at 550 °C. The strong interlayer interaction and high surface migration of adatoms on *c*-sapphire enable the assembly of 1D nanowires into 2D nanosheet under 600 °C. Based on the characterization results demonstrated, we propose the van der Waals growth mechanism of 1D nanowires and 2D nanosheets. Moreover, the visible-light photocatalytic activity of 1D nanowires and 2D nanosheets was examined. Both 1D and 2D GaTe nanostructures exhibit visible-light active photocatalytic activity, suggesting that the GaTe nanostructures may be promising materials for visible light photocatalytic applications.

## 1. Introduction

Owing to their fascinating physical and chemical properties, the emergence of two-dimensional (2D) semiconductor materials has attracted considerable interest in the field of optoelectronics, nanoelectronics, chemical sensors, photocatalysts, and energy-related applications [1,2,3,4]. The 2D materials exhibit unique properties such as high mobility of charge carriers, large surface area, dangling bond-free surface, and high mechanical strength. The large surface areas provide more active sites for photocatalytic reaction to occur, and the unique 2D geometry provides a shorter migration distance for photogenerated carriers to transport, further prevent recombination of photo-generated charge carriers [5]. As a result, the 2D materials are potentially useful for potential optoelectronics and photocatalysts. For example, transition-metal dichalcogenides (MoS_2_, and WS_2_) [6,7], graphitic carbon nitrides (g-C_3_N_4_) [8], Bi_2_MoO_6_ [9,10], and phosphorenes [11] have been demonstrated as potential visible light active photocatalysts for water splitting and organic synthesis. Developing novel 2D photocatalysts that can efficiently utilize solar spectrum to dissociate water and produce hydrogen is highly desirable.

The monochalcogenides (MX, M = Ga and In, X = S, Se, and Te), such as GaS, GaSe, GaTe, InSe, and InTe, have been extensively studied due to their potential application in next-generation electronic and optoelectronic devices [12,13,14,15,16]. The layers are composed of two metal and two chalcogen atoms, forming a X-M-M-X assembly perpendicular to the layer. The monolayers are stacked by van der Waals forces to construct layered structures. While most monochalcogenides exhibit hexagonal structures (GaS, GaSe, InS, and InSe), gallium telluride (GaTe) exhibit a lower symmetry with a monoclinic structure and crystallizes in a complicated structure than those of other MX materials [17]. GaTe exhibits high carrier mobility, long carrier lifetime among the monochalcogenides. An indirect band gap occurs when M-M bonds are oriented normal to the layer planes, such as GaS and GaSe. One-third of the M-M bonds are parallel to the layer planes, which suggests a direct band gap nature of GaTe [18]. The GaTe is a p-type semiconductor with a direct band gap of 1.65 eV for all thickness, which indicates that GaTe may be potentially useful in optoelectronic and photocatalytic applications. Recent reports demonstrated high-performance photodetectors with high photoresponsivity by various GaTe nanostructures [13,14,19,20,21].

The synthesis of GaTe 2D nanostructures normally accomplished by micromechanical exfoliation of bulk crystals synthesized by Bridgman method in the literature [13,14,19]. Although the 2D nanostructures are high-quality and exhibit superior physical properties and high-performance optoelectronic devices were reported, the morphology cannot be well controlled and the size of the sample is normally very small. On the other hand, the bottom-up method including chemical vapor deposition (CVD) and physical vapor transport (PVT) of synthesizing 2D nanostructures, offers advantages in control of morphologies, layer thickness, and crystallinity, which alters material properties and is highly desired for practical applications [22,23,24,25]. To date, a few reports are demonstrating the vapor phase deposition of various one-dimensional (1D) and 2D GaTe nanostructures including nanoflakes, nanosheets, and nanowires. Yu et al. first reported the synthesis of 1D GaTe nanowires on Si by vapor-liquid-solid (VLS) mechanism using CVD. The synthesis of 2D GaTe nanosheets by CVD and the fabrication of photodetector was also demonstrated [26]. Cai et al. investigated the effect of different substrates including GaAs (111), Si (111), and *c*-sapphire on the PVT growth of GaTe 1D and 2D nanostructures [27]. It was found that the morphology of the GaTe nanostructures strongly depends on the substrate: GaTe 1D nanowires were obtained on GaAs (111) and Si (111) due to the epitaxial relationship between GaTe and different substrate, while 2D GaTe nanoflakes grow randomly on the *c*-sapphire substrate via the van der Waals epitaxy growth mechanism. Instead of isotropic growth, the preferable growth along [010] of GaTe is attributed to the highly anisotropic monoclinic crystal structure. Therefore, the selective growth of GaTe nanostructures with controlled morphology and aspect ratio is crucial since the fundamental chemical and physical properties may be very different between 1D and 2D nanostructures, which may lead to different applications. Therefore, the morphology-controlled synthesis of 1D and 2D GaTe nanostructures by vapor phase deposition remains challenging.

In this study, we report the morphology-controlled synthesis of 1D and 2D GaTe nanostructures through the simple PVT approach. By adjusting proper growth temperature and growth time, the morphology-controlled synthesis of 1D GaTe nanowires and 2D GaTe nanosheets were demonstrated. The samples were characterized using scanning electron microscopy (SEM), transmission electron microscopy (TEM), micro-Raman, micro-PL, and UV/VIS spectroscopy. The surface morphology, crystal structure, phonon vibration modes, and optical property of samples were examined and studied. Based on the characterization results, we propose the van der Waals growth mechanism of 1D GaTe nanowires and 2D nanosheets. Moreover, the visible-light photocatalytic activity of 1D and 2D GaTe nanostructures was examined. Both 1D and 2D GaTe nanostructures show visible-light active photocatalytic activity, suggesting that the GaTe nanostructures may be promising materials for potential visible light photocatalytic applications.

## 2. Experiment Section

### 2.1. Morphology-Controlled Synthesis of 1D and 2D GaTe Nanostructures

The samples were prepared by a simple PVT method where 0.5 g of gallium (99.999%, Alfa Aesar Inc., Ward Hill, MA, USA) and 0.2 g of tellurium powder (99.999%, Alfa Aesar Inc., Ward Hill, MA, USA) were placed in alumina crucibles in a 2-inch quartz tube in a furnace (Thermo Fisher Scientific Inc., Waltham, MA, USA). The evaporation temperature of gallium (~900 °C), tellurium (~400 °C), and the growth temperature of samples (~500, 550, and 600 °C) were controlled by the different locations of heating zones. The *c*-sapphire substrates were cleaned by acetone, methanol, and deionized water, placed downstream from the source materials at the position of desired growth temperatures. The argon was introduced into a quartz tube and purged 3 times and flowed at a rate of 20 sccm to maintain an inert environment during heating up to 900 °C at a rate of 10 °C/min. Once the furnace reached 900 °C, the flow rate of argon was increased to a rate of 100 sccm and a base pressure of 0.5 Torr of the tube was evacuated by a mechanical pump. The typical growth time was 1 h. After growth, the samples were cooled under ambient argon at a flow rate of 20 sccm. The morphology-controlled synthesis of 1D GaTe nanowires could be obtained at a growth temperature of 550 °C, while the 2D GaTe nanosheets were synthesized under 600 °C.

### 2.2. Sample Characterization

The surface morphologies of samples were observed by scanning electron microscopy (SEM) images on a Hitachi S-3400N microscope (Hitachi Ltd., Tokyo, Japan). The structural properties of samples were characterized by X-ray diffraction (XRD) on a Bruker D8 Advance diffractometer (Bruker Inc., Billerica, MA, USA). A transmission electron microscopy (TEM, JEM-3010, JEOL Ltd., Tokyo, Japan) was used to verify the crystal structure, growth direction, and assembly of nanostructures. A micro-Raman scattering system (Renishaw 1000B, Renishaw Ltd., Gloucestershire, UK) was used to record micro-Raman and micro-photoluminescence (micro-PL) spectra of samples under 532 nm excitation at room temperature. The absorption spectra of samples were obtained using ultraviolet–visible spectroscopy (UV/vis, U-3900, Hitachi Ltd., Japan).

### 2.3. Characterization of Visible-Light Photocatalytic Activity

The visible-light photocatalytic activity of the samples was estimated by recording the photo-decomposition rate of methyl blue (MB) solution (1 × 10^−5^ M) under visible light irradiation. The as-grown samples were added to 10 mL of MB solution in a quartz plate-covered beaker for the photocatalytic test. The solutions were placed in the dark for 30 min to establish the adsorption/desorption equilibrium. A 500 W Xe arc lamp with a UV cutoff filter (λ > 420 nm) was used as the visible light irradiation source. The characteristic absorption of MB (664 nm) was measured as an indicator of the photodecomposition of MB using an ultraviolet–visible spectrometer (UV/vis, Hitachi U-3900).

## 3. Results and Discussion

### 3.1. Morphology-Controlled Synthesis of GaTe Nanowires and Nanosheets

We synthesize GaTe nanostructures using a simple PVT process as described in the experimental section. Although most recent studies report the physical-vapor-deposition (PVD) growth of GaTe layer samples using GaTe powder as the precursor [20,27,28]. Considering to the high melting point of GaTe (824 °C), using the low melting point materials: gallium (mp: 30 °C) and tellurium (mp: 450 °C) as evaporation source not only provides a steady supply of Ga and Te vapor pressure but also allows the precise morphology control of the growth of GaTe 1D nanowires and 2D nanosheets.

**Figure 1:**
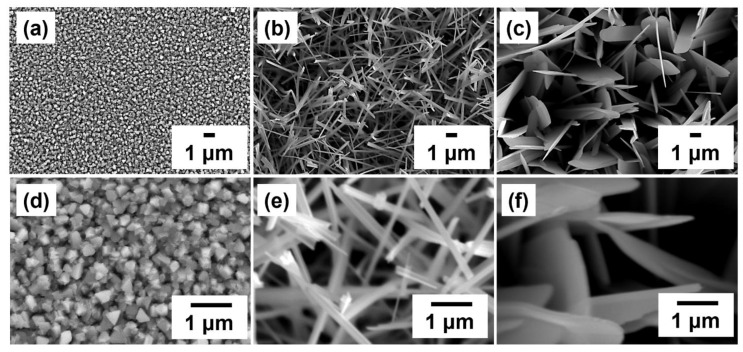
Top-view SEM images of sample grown on *c*-sapphire at different growth temperatures: (**a**) 500 °C, (**b**) 550 °C, and (**c**) 600 °C with 60 min growth time. The high-magnification SEM images ((**d**) 500 °C, (**e**) 550 °C, and (**f**) 600 °C) clearly show the surface morphology of the sample change with growth temperature.

In order to optimize the growth conditions of GaTe nanostructures, first we examine the effects of different growth temperatures on the surface morphologies and phase composition of the as-grown samples. The samples were grown at three different growth temperatures (500, 550, and 600 °C) for 1 h, and the surface morphologies of samples were characterized by SEM as illustrated in Figure 1a–c. It is clearly observed that the morphologies are grown ranged from the high density of rectangular and triangular crystals (500 °C) to 1D nanowires (550 °C) and two-dimensional nanosheets (600 °C) as the growth temperature increases. The rectangular crystals were in the size of 200–300 nm in high density at the growth temperature of 500 °C as shown in Figure 1d. It reveals that different crystal structures and compositions exhibit on the substrate. For the sample grown at 550 °C, the nanowires with diameters of 100–300 nm and lengths 1–3 μm were obtained (Figure 1e). The nanosheets with 1–1.5 μm width and 4–5 μm in lengths were observed on the sample grown at 600 °C (Figure 1f). It is concluded that the diameters of GaTe nanowires and the lateral size of GaTe nanosheets can be controlled by adjusting the growth time. The distribution of GaTe nanostructures is random because the GaTe is bonded to the *c*-sapphire substrate by van der Waals force without forming true chemical bonds. It is clearly shown that the morphology-controlled synthesis of GaTe nanowires and GaTe nanosheets can be achieved by adjusting the growth temperature in a facile thermal evaporation setup.

### 3.2. Morphologies Evolution with Growth Time

**Figure 2:**
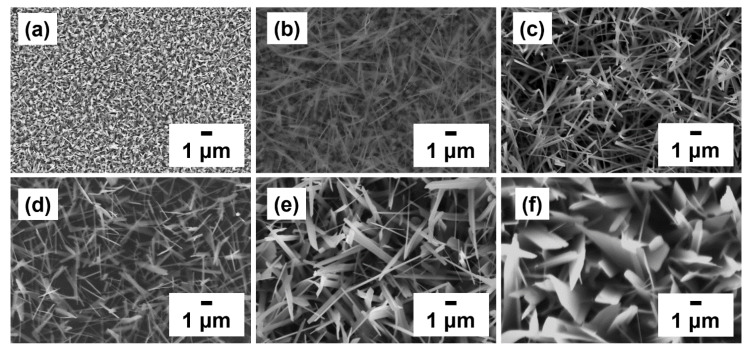
Top-view SEM images of gallium telluride (GaTe) nanowires and nanosheets sample grown on *c*-sapphire with different growth times: GaTe nanowires (**a**) 5 min, (**b**) 20 min, and (**c**) 60 min and GaTe nanosheets: (**d**) 5 min, (**e**) 20 min, and (**f**) 60 min.

The effect of growth time (5, 20, and 60 min) on the surface morphologies of the sample was studied at 550 and 600 °C. Moreover, the growth mechanism of GaTe nanowires and nanosheets of these two distinct nanostructures were further investigated. Figure 2 shows the GaTe nanowires (a–c) and nanosheets (d–f) grown on *c*-sapphire with the different growth period. The initial growth sample of GaTe nanowires consists of a large amount of rectangular crystals accompany with a few 1D nanowires. When the growth time increased to 20 min, a large amount of GaTe nanowires was observed. Eventually, when the growth time further increased to 60 min, the GaTe nanowires with slightly larger diameters and longer lengths were obtained. In contrast, the surface morphology of the GaTe nanosheet appears to consist of well-defined 2D nanostructures with slightly increased lateral size with growth time. Apparently, the growth temperature is a key factor to control the sample morphology of GaTe nanostructures. A steady supply partial pressure of Ga and Te is essential toward the growth of high-density GaTe nanostructures, and the morphology can be adjusted by controlling growth temperature and growth time.

**Figure 3:**
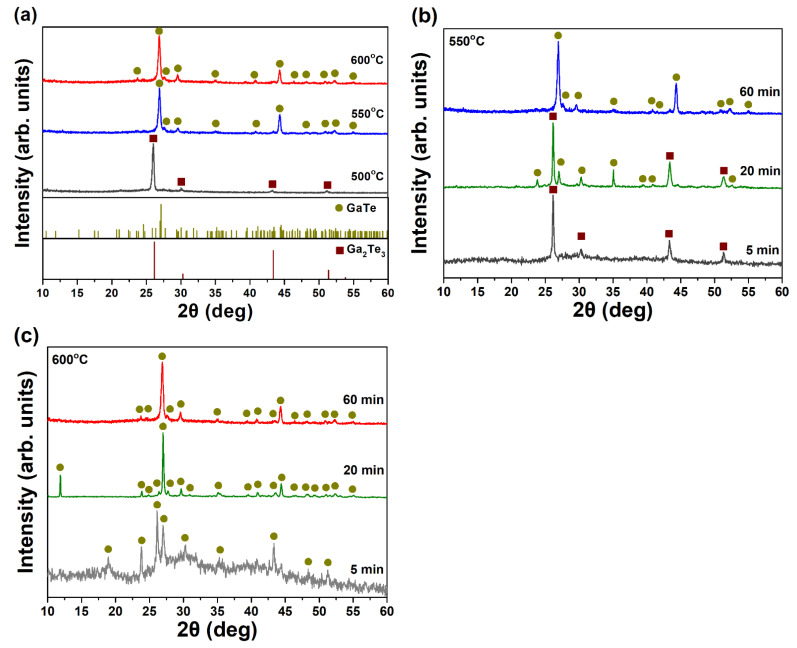
The XRD patterns of samples (**a**) grown at different growth temperatures (500, 550, and 600 °C) for 1 h, (**b**) grown at 550 °C with different growth time (5, 20, and 60 min), and (**c**) grown at 600 °C with different growth time (5, 20, and 60 min).

To further confirm the phase purity and the temperature window for the growth of GaTe nanostructures, the as-grown samples were characterized by XRD. The XRD patterns of samples deposited at different growth temperature for 1 h are shown in Figure 3a. For the sample is deposited at 500 °C, the diffraction peaks were indexed as cubic Ga_2_Te_3_ phase (brown squares, JCPDS 35-1490), which is consisted of the observed high density of rectangular crystals from SEM (Figure 1a). As the deposition temperature increased to 550 and 600 °C, both of the diffraction patterns were indexed as monoclinic GaTe phase (dark yellow circles, JCPDS 33-0571) with high phase purity, suggesting the GaTe phase can be synthesized at growth temperature higher than 550 °C. The strong diffraction peak of (020) located at 2θ value of 44 indicates a preferred [020] out-of-plane orientation in the GaTe nanowires samples. The structural characterization of samples with different growth periods under 550 and 600 °C were also investigated by XRD, which are illustrated in Figure 3b,c, respectively. The phase composition of the sample grown at 550 °C consisted of mixed GaTe and Ga_2_Te_3_ phases, suggesting that the growth of GaTe nanowires begin with the initial nucleation of Ga_2_Te_3_ nanocrystals on the *c*-sapphire substrate at 550 °C. The phase composition of samples remains the same monoclinic structure for all samples with different growth time deposited at 600 °C, suggesting that the nucleation of GaTe occurs immediately on the substrate at initial growth stages under the higher temperature. The results are consistent with the reported crystallization temperature ~450 °C of Ga_2_Te_3_ [29,30].

**Figure 4:**
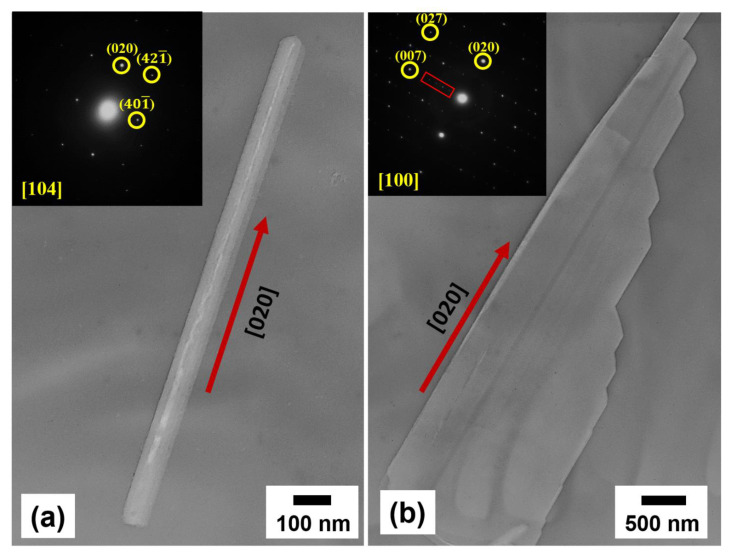

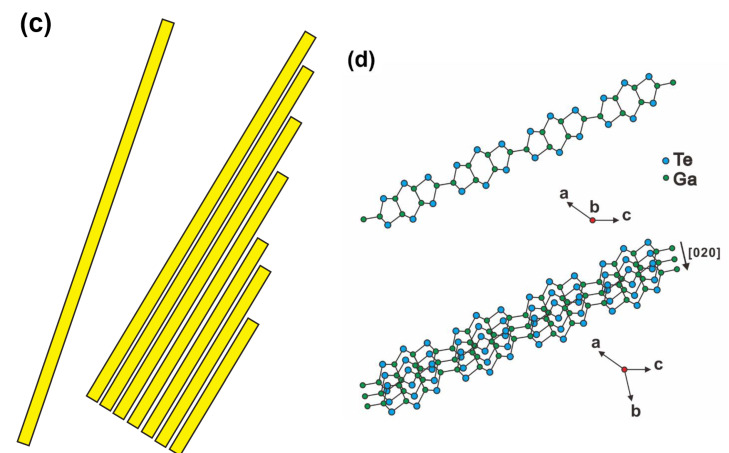
(**a**) TEM image of single GaTe nanowire. Inset shows selected area electron diffraction (SAED) patterns revealing its single-crystalline monoclinic structure and a [020] growth direction. (**b**) TEM image of single GaTe nanosheet. Inset shows SAED patterns with extra spots along the short axis of nanosheets. (**c**) Schematic illustration of assembling GaTe nanowires into nanosheets. (**d**) Schematic illustration of single GaTe layer stacking into GaTe nanowire along the b-axis.

The TEM was utilized to perform structural characterization of single GaTe nanowire and GaTe nanosheet in more detail. Figure 4a shows the TEM image of a single GaTe nanowire with a diameter of 60 nm and 1.28 μm in length. The nanowire reveals single-crystalline monoclinic structure with a [020] growth direction confirmed by selected area electron diffraction (SAED) pattern. A representative TEM image of a single GaTe nanosheet is shown in Figure 4b, revealing the irregular edge and the lamellar morphology. The inset shows SAED pattern taken from a nanosheet, the spot pattern can be well indexed to monoclinic GaTe, and a [020] growth direction along the long axis, which is also observed from a single GaTe nanowire. Different from the single nanowire, additional satellite spots with equal spacing (region marked by a red square) along the short axis were also observed in the nanosheet. The result is attributed to a larger d-spacing layer stacking along the short axis with a monoclinic structure, indicating that the single nanosheet is assembled of multiple nanowires by interlayer interaction. The schematic illustration of assembling GaTe nanowire into nanosheet is displayed in Figure 4c. Based on the above TEM results and the highly anisotropic structure of GaTe, it is concluded that the GaTe layer stacking into GaTe nanowire along the b-axis as illustrated in Figure 4d. The anisotropic growth of GaTe nanostructures with preferable b-axis growth direction on *c*-sapphire was suggested by a series of atomic chains along the [010] direction reported in the literature [27]. Obviously, the GaTe nanosheet was assembled by GaTe nanowire only under a higher growth temperature, suggesting that the growth kinetics is different between 550 and 600 °C. Under the higher growth temperature, the surface adsorbed atoms migrate more easily on the surface, the surface reaction is faster and the interlayer interaction is more likely to occur, thus the stacking of 2D GaTe nanostructures is more favorable.

Based on the above results, we propose the following growth model for GaTe nanostructures growth on the *c*-sapphire substrate. The difference in surface morphologies of samples is mainly attributed to the different phase stability, higher surface mobility of surface adsorbed Ga and Te atoms, and the assembly of nanowires into nanosheets under a higher growth temperature. The effect of surface reaction rate and interlayer interaction are the two possible reasons which may alter the 1D and 2D growth of GaTe nanostructures. The initial nucleation sites can be formed during direct deposition of Ga and Te precursor in gas phase or solid phase via surface migration on the *c*-sapphire substrate. The temperature-dependent surface migration coefficient (D) of adatoms on the substrate is described by:

$$D=D_{0}e^{\frac{-E_{m}}{kT}},$$

where $D_{0}$ is the pre-factor, which is independent of temperature, $E_{m}$ is the surface atom migration energy barrier, *k* is the Boltzmann constant, and *T* is the substrate temperature. The formation nucleation site and phase formation are highly related to surface reaction, and the surface reaction rate is strongly correlated to the growth temperature. The surface migration coefficient of adatoms under the higher growth temperature is higher than those under the lower growth temperature, the surface reaction rate is higher, and the interlayer interaction is stronger, as a consequence, 2D GaTe nanosheets grow immediately on the *c*-sapphire surface at a higher growth temperature. For samples deposited at a lower growth temperature, the insufficient surface migration of adatoms, low surface reaction rate, and a weaker interlayer interaction lead to preferential growth of 1D GaTe nanowires.

### 3.3. Optical Properties of GaTe Nanowires and Nanosheets

**Figure 5:**
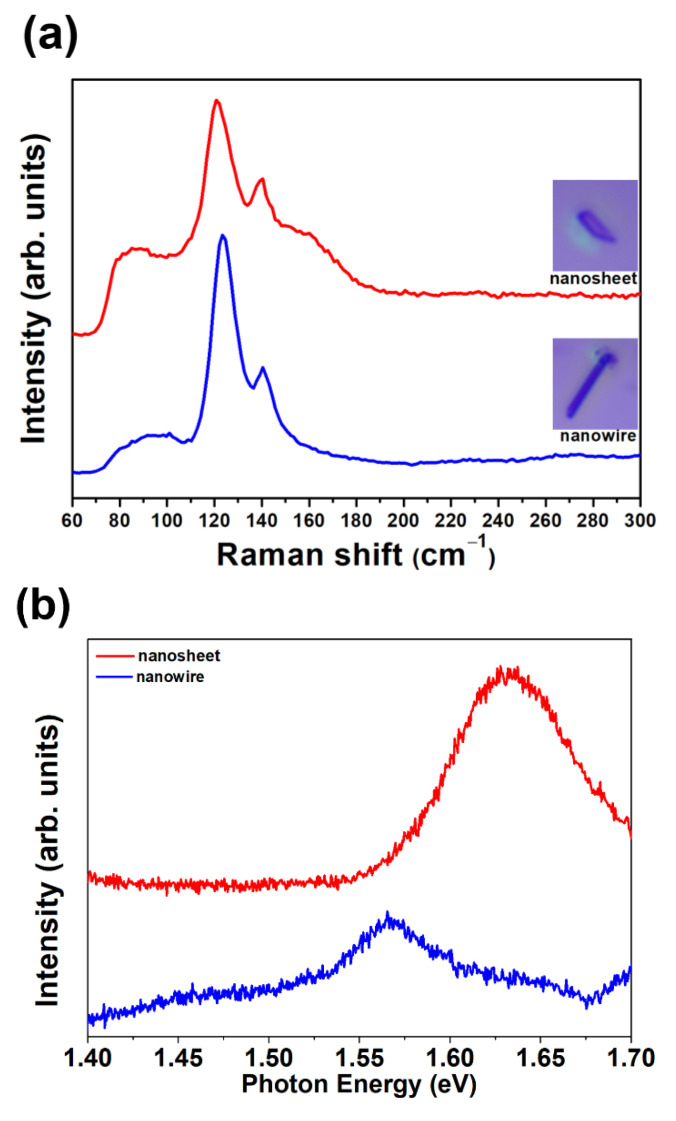

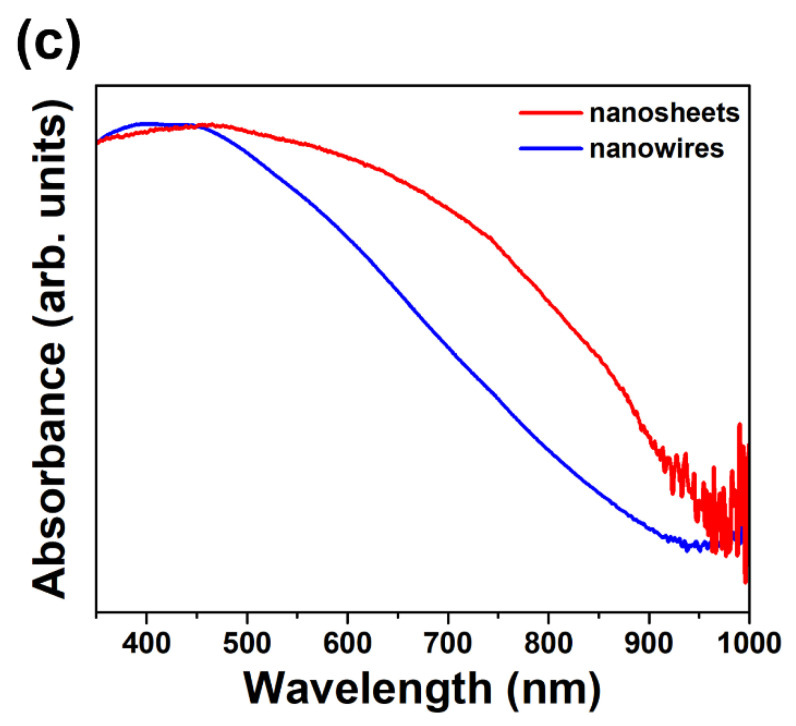
(**a**) Raman spectra of single GaTe nanowire and nanosheet. (**b**) PL spectra of single GaTe nanowire and nanosheet. (**c**) UV-visible absorption spectra of GaTe nanowires and nanosheets.

The optical properties of GaTe nanowires and nanosheets were characterized by micro-Raman, micro-PL, and UV–visible absorption spectra. Figure 5a shows the micro-Raman spectra of a single GaTe nanosheet and GaTe nanowire, the inset shows the optical micrograph of typical single nanostructure measured. The vibrational modes at ~89, ~91, 123, and 141 cm^−1^ are observed in a single GaTe nanowire. The peaks at ~123 and ~141 cm^−1^ are attributed to interlayer double resonant B_g_ and A_u_ modes. The peaks at ~89 and ~91 cm^−1^ correspond to A_g_ and B_u_ vibration mode, respectively. Different Raman vibrational modes at ~90, ~92, 120, 140, and 160 cm^−1^ are observed in single GaTe nanosheet. Additional lateral vibration peak at ~160 cm^−1^ (B_g_) was observed in GaTe nanosheet, indicating that different interlayer van der Waals interactions exist between 1D and 2D samples. Moreover, a blue shift is observed in the GaTe nanowire, possibly due to a reduced lattice constant and stiffening in the vibration mode [31]. The observed vibration modes precisely match with reported peaks observed from monoclinic GaTe layered samples grown by a vapor deposition method, and no secondary phase (Ga_2_Te_3_, Ga_2_O_3_, or TeO_2_) was detected, which is in good agreement with our characterization results reported previously [18,27].

The PL spectra of single GaTe nanowire and nanosheet were measured at room temperature as displayed in Figure 5b. A broad emission band centered at 1.64 eV were observed in GaTe nanosheet, indicating that the emission originates from different radiative recombination centers among band gap. The emission band (FWHM~100 meV) centered at 1.65 eV (A), corresponding to both band to band transitions and various intrinsic defect-related transitions such as Ga vacancy (V_Ga_), Te vacancy (V_Te_), and Te-on-Ga antisite (Te_Ga_). The V_Ga_ and the Te_Ga_ are acceptors, while V_Te_ is a donor [19,32,33]. The direct band to band radiative recombination of photogenerated electrons and holes of the GaTe gives rise to emission at ~1.65 eV at room temperature. The different intrinsic or structural defects may act as shallow sensitizing centers or deep recombination centers, resulting in various defect-related emission below optical band gap, which has been reported previously [21,27]. Obviously, the emission from GaTe nanosheet is composed of mainly direct band to band emission and partial shallow defect-related transition. In contrast, the GaTe nanowire shows a weak band to band emission and an additional emission peak at ~1.56 eV. The weak band to band emission of single GaTe nanowire is possible due to their large surface area, which has also been reported in the literature, and the emission peak centered at ~1.56 eV originates from recombination of photogenerated electron–hole pairs at shallow sensitizing centers, consisting of defects such as V_Ga_ and Te_Ga_, which have been verified by first-principles calculations [19]. It is concluded that the different types of structural defects exist in GaTe nanowire and nanosheets, the different structural defects may originate from the different interlayer interaction of sample. Figure 5c shows the UV/VIS absorption spectra of GaTe nanowires and nanosheets. With the optical band gap energy of 1.65 eV, GaTe exhibits strong absorption in the visible light and transmittance in the infrared region. The GaTe nanosheets show a stronger visible light absorption compared with those of GaTe nanowires, possibly due to the unique 2D geometry and strong interlayer coupling effect of 2D structures [34].

### 3.4. Visible-Light Active Photocatalytic Activity

**Figure 6:**
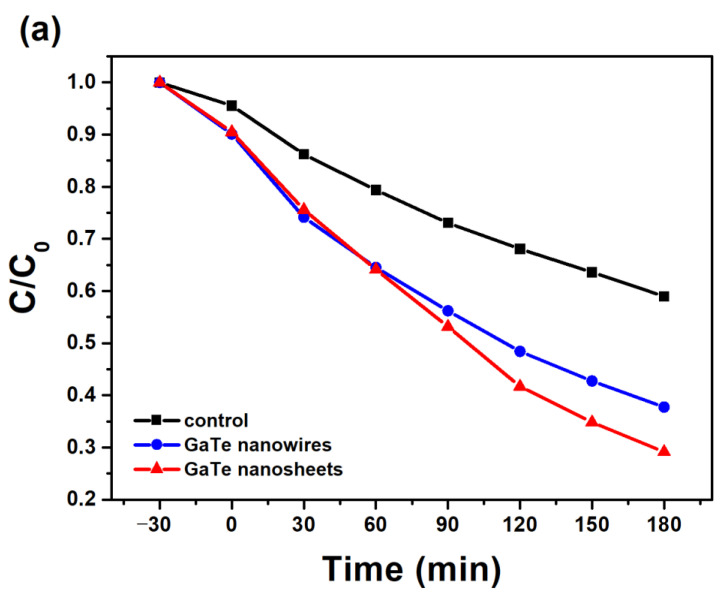

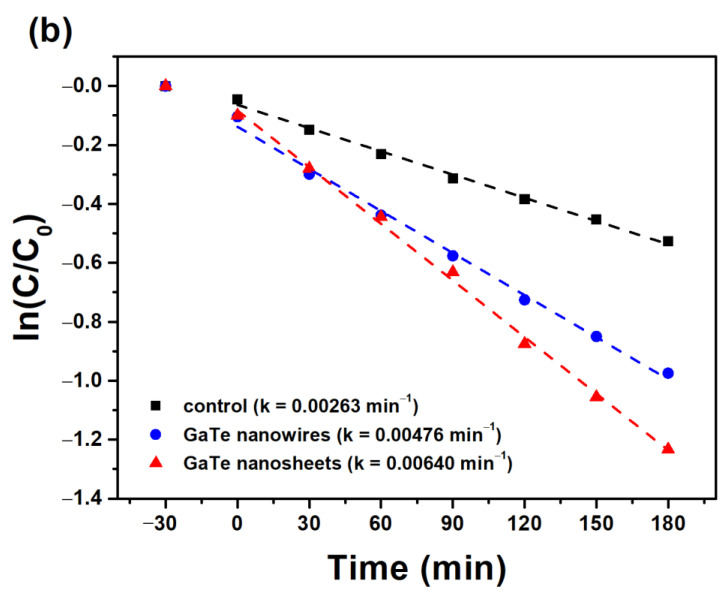
(**a**) Visible-light photocatalytic degradation of methyl blue (MB) solution of GaTe nanowires and nanosheets. (**b**) Visible-light photocatalytic rates of GaTe nanowires and nanosheets.

Owing to its moderate and direct band gap of 1.65 eV in bulk as well as in single layer forms, GaTe may exhibit photocatalytic properties. Recently, the photocatalytic hydrogen evolution reaction (HER) of GaTe by both theoretical and experimental studies has been reported [35]. Here, we examine the visible-light active photocatalytic activity of GaTe nanowires and nanosheets using photodegradation of MB solution under visible light illumination. Figure 6a shows the visible-light photocatalytic degradation of MB solution of GaTe nanowires and nanosheets samples. Although the amount of photocatalysts (~1–2 mg) are relatively small, both samples exhibit considerable photocatalytic activity under visible light illumination. The photodecomposition reaction rate constant was calculated by the pseudo-first order reaction rate law as shown in Figure 6b. The reaction rate constants of GaTe nanowires and GaTe nanosheets are 0.00476 and 0.0064 min^−1^, respectively. It is clear that the GaTe nanosheets show a slightly higher photocatalytic activity than that of GaTe nanowires. It has been reported that the different sizes, morphologies, number of layers of 2D materials, cause different photocatalytic activity [36,37]. The photocatalytic activity of GaTe is associated with the hydroxyl radical species (·OH) and peroxide ($\cdot O_{2}^{-}$), created by surface reactions with photogenerated electron–hole pairs as proposed as follows:

$$\mathrm{GaTe}+h\upsilon \to \mathrm{GaTe}\left(e^{-}+h^{+}\right)\;\mathrm{GaTe}\left(e^{-}\right)+O_{2}\to \mathrm{GaTe}+\cdot O_{2}^{-}\;\mathrm{GaTe}\left(h^{+}\right)+{\mathrm{OH}}^{-}\to \mathrm{GaTe}+\cdot \mathrm{OH}\;\cdot O_{2}^{-}+\mathrm{MB}\to {\mathrm{CO}}_{2}+H_{2}O\;\cdot \mathrm{OH}+\mathrm{MB}\to {\mathrm{CO}}_{2}+H_{2}O.$$

The observed photocatalytic activity is mainly attributed to the large surface area, the moderate and direct band gap, and high visible light absorption of multilayer GaTe nanostructures. The large surface area of GaTe nanowires and nanosheets provides more surface sites for photocatalytic reaction to occur, the moderate band gap, and high visible light absorption of multilayer GaTe nanostructures, ensuring the generation of electron–hole pairs by visible light illumination more efficiently during the reaction. The results suggest that the GaTe nanostructures may be promising materials for potential visible light photocatalytic applications such as solar water splitting, carbon dioxide reduction, and degradation of organic pollutants.

## 4. Conclusions

The 1D GaTe nanowires and 2D nanosheets are successfully grown on the *c*-sapphire substrate using a simple PVT method. With controlled growth temperature and growth time, single-crystal monoclinic 1D GaTe nanowires and 2D GaTe nanosheets can be selectively synthesized under 550 and 600 °C, respectively. The growth temperature is a key factor to control sample morphology. The strong interlayer interaction and high surface migration of adatoms enable the assembly of nanowires into nanosheets under 600 °C. The effect of growth parameter on phase composition, surface morphology, and growth mechanism of sample are discussed in detail. The samples exhibit different structural defects, possibly due to the different interlayer interactions of samples. In addition, both 1D and 2D GaTe nanostructures exhibit visible light photocatalytic activity, suggesting that the GaTe nanostructures may be promising materials for potential visible light photocatalytic applications.

## Data Availability

The data presented in this study are available within this article. Further inquiries may be directed to the authors.
